# Indicanuriax

**Published:** 1913-11

**Authors:** 


Indicanuriax. Gustav Baar, Portland, Ore., *Northwest Med.,* July, 1913. Constipated cases showed a positive test 736 times, negative 820 times, those without constipation, positive 2600 times, negative 2503 times. There were 2092 cases altogether. There seemed to be no necessary coincidence with intestinal indol. (Note. There is probably more or less indol in all fæces, but the test is not very delicate, not nearly so delicate as that for indican in the urine. Moreover, absorption need not necessarily correspond to absorption.) Indican of metabolic origin is regarded as improbable. Indican due to decomposition of retained pus, fœtus, gangrenous matter, etc., is considered rare. Exclusive lacto-farinaceous diet, vegetable diet or sour milk or lacto-bacillin did not influence the indican. (Note. In a personal experience we have found, usually, a moderate though not very rapid response to such theoretic indications. Still, it is not uncommon to find proteid decomposition coincident with marked acid fermentation and gas production in the intestine.) The author speaks of the plausible argument for an action of hyperchlorhydria to prevent or cause indicanuria, and, in an experience with 276 cases with an acidity (total?) about 70 degrees, found a positive test 560 times and negative 550 times, which corresponds with our own experience and probably with that of everyone who "experiments, not thinks." The article contains many other interesting details.

